# Co-production between long-term care units and voluntary organisations in Norwegian municipalities: a theoretical discussion and empirical analysis

**DOI:** 10.1017/S1463423620000341

**Published:** 2020-09-15

**Authors:** Nina Beate Andfossen

**Affiliations:** NTNU Norwegian University of Science and Technology, Faculty of Medicine and Health Sciences (MH), Department of Health Sciences in Gjøvik, Centre for Care Research, Gjøvik, Norway; Inland Norway University of Applied Sciences, Centre for Innovation in Services, Lillehammer, Norway

**Keywords:** collaborative innovation, co-production, public care services, voluntary work

## Abstract

**Background:**

In Norway, due to demographic challenges with an ageing population and lower fertility rates, current government policies have encouraged municipalities and volunteers to collaborate. Moreover, present policies recommend an increase in volunteer activities within care services. Co-production is advocated as a functional and innovative method of activating resources when citizens and public employees interact in the care sector.

**Method:**

This study has scrutinised ongoing volunteer activities in nursing homes and home care facilities by utilising the results from a survey targeting employees in public care services.

**Aim:**

The aim has been to identify the extent to which long-term care units (LTC units) in Norwegian municipalities and voluntary organisations collaborate in the coordination of volunteer activities at the local level by answering the following research questions: when LTC units and voluntary organisations collaborate in coordinating voluntary activities within caring services: are they sharing tasks, dividing the tasks between them or both?

**Findings:**

The results show that LTC units often coordinate volunteer activities that correspond to statutory public care services. Additionally, LTC units also contribute considerably in coordinating other volunteer activities, either alone or to a small extent in collaboration with voluntary organisations. This limited task sharing when coordinating volunteer activities in municipal care services can be seen as a suboptimal way of using the resources. Hence, a large part of this paper concerns a discussion of the theory of co-production in public care services, drawing on the findings of the survey.

## Background

Due to demographic challenges with an ageing population and lower fertility rates, the World Health Organisation (WHO, 2015) predicts that actions in future service delivery will inevitably require more resources. In the quest to accommodate these challenges, Western governments have put increasing focus on volunteer activities within primary care services by seeking new ways to utilise collaboration with the third sector and hence increase voluntary unpaid work in these services.

Pollitt and Bouckaert (2017) state that ‘conceptually identical, or at least similar, reforms develop differently in one national (or sectoral or local) context as compared with another’ (p. 46). Additionally, Voorberg et al. (2017) illustrate that ‘state and governance traditions can explain why governments respond differently to similar challenges’ (p. 371). Norway is used as the research context for this study, representing a Nordic, or social-democratic regime, which, according to Esping-Andersen (1990), is characterised by a strong public sector. Volunteer activities in Norway are carried out locally in the municipalities. This study focuses on voluntary activities carried out in care services within the municipalities.

Although acknowledging the variety of volunteer engagements and different types of volunteers both within and outside of voluntary organisations (Andfossen, 2016, 2019), this paper relates to a commonly used definition of voluntary work as ‘the work a person does within voluntary organisations for others than family and close friends without receiving regular payment for it’ (Wollebæk and Sivesind, 2010). In Norway, current policies recommend an increase in volunteer activities within care services. Additionally, municipalities and volunteers are encouraged to collaborate, and co-production is advocated as a functional and innovative method of activating resources when citizens and public employees interact in the care sector (Ministry of Health and Care Services, 2013). Moreover, the opportunities for innovation are expected to emerge in the interstices between public services and civil society (Ministry of Health and Care Services, 2011b). Data from 2010 reveal that more than half of the Norwegian municipalities had agreements with voluntary organisations concerning the provision of volunteer activities (Loga, 2012). Furthermore, 64% of the agreements in the public sector occur in the care sector (KS, 2010; Loga, 2012). Nevertheless, data from 80% of Norwegian municipalities indicate that they do not have (69%) or do not know (11%) whether they have a volunteer policy (Loga, 2012). Agreements and ongoing activities confirm that there is a collaboration between municipalities and volunteers, but the collaboration in organising and performing activities seems to be insufficiently linked to an overall local municipal volunteer policy.

Previous third sector research has tended to ignore the local level where many governmental and third sector agreements are implemented, focusing instead on formal agreements at the national level (Pestoff et al., 2006). Since clear policies for collaboration between public care services and voluntary organisations seem to be lacking, one can envision at least two forms of collaboration. One form of collaboration is a sharing of tasks between public and voluntary actors. Another form of collaboration is a division of tasks. Concerning *the first* mentioned, collaboration on tasks can stimulate heterogeneity and bring in new ideas (Sørensen and Torfing, 2011b). However, it might also bring about a competitive situation, where both parties engage in tasks included in the other party’s ‘strong areas’. Roberts (2000) describes competitive strategies as one of three coping strategies when dealing with wicked problems. Today, municipal care services are loaded with wicked problems where many actors and competing interests in a complex organisation are engaged in problem-solving (Tortzen, 2019), which may lead to a suboptimal way of using the resources. Concerning *the second form* of collaboration – division of tasks – it is reasonable to expect that the public care sector carries out its own statutory tasks and that voluntary organisations focus on non-statutory tasks. This can be seen as a way of exploiting the strengths of both parties and limiting the use of resources. However, it might also hamper the adoption of innovative forms of collaboration that can contribute to mobilising resources, such as an increased number of volunteers.

By examining ongoing volunteer activities in nursing homes and home care facilities in Norway, this study aims to identify the extent to which long-term care units (LTC units) and voluntary organisations collaborate in the coordination of volunteer activities at the municipal, local level. This paper contributes theoretically and empirically to the field by discussing the results in light of a theoretical framework of co-production with the aim of extending the understanding of co-production and collaboration among municipal care services and voluntary organisations. Furthermore, the discussion will elucidate the potential for collaborative innovation. The paper aims to answer the following research questions: *When LTC units and voluntary organisations collaborate in coordinating voluntary activities within caring services: are they sharing tasks, dividing the tasks between them or both?* The next section will outline voluntary work in the Norwegian public care context in more detail and illustrate the actual situation for co-production in such a regime.

## Voluntary work in a Norwegian public care context

In Norway, the government has stated that it wants multiple actors to participate and collaborate in the delivery of care services (Ministry of Health and Care Services, 2013). In that context, volunteers are portrayed as potentially important actors.

The health and care services in Norway are divided into different areas of responsibility. As a part of this highly developed and decentralised public primary healthcare service system, the overall responsibility for providing and managing care services rests on the municipalities. The health and long-term care sector in Norway is fairly large, and most care services are provided locally within the municipality, primarily as institutional services like nursing homes and as home care services. Nursing homes and home care services are the largest cost area for the municipalities – as of 2017, this constituted 310 000 person years (Leknes et al., 2019; Ministry of Health and Care Services, 2011a).

Norwegian municipalities are required to cater for the diversity of needs among the care receivers. The Health and Care Services Act and the Regulation on quality in care services state that the municipalities shall have written procedures that specify the tasks and content of the care services in order to meet the care recipients’ basic needs (Ministry of Health and Care Services, 2003, 2011a). Basic needs include physiological needs (like food and drink) and social needs (like interaction, togetherness and activity). This also includes personal hygiene and necessary medical examinations and treatment as well as rehabilitation and care tailored to each individual’s needs. With respect to these comprehensive tasks, a white paper states that the greatest challenge in care services today is to ensure the provision of meals and activities and to meet social and cultural needs (Ministry of Health and Care Services, 2006). Moreover, healthcare professionals in elderly care services recognise the growing importance of focusing on activation and socialising (Hillestad and Tessem, 2015). However, they experience that there is insufficient time within service provision to satisfy these needs (Hillestad and Tessem, 2015).

Independently of the vast public care sector in Norway, organised volunteers have a long tradition of contributing towards long-term care services (Lorentzen and Selle, 2000). At the same time, cross-national differences are registered in terms of the *nature* and *scope* of voluntary contributions (Ferreira, 2006; Musick and Wilson, 2008). Concerning *the scope*, despite an overall increase in the prevalence of voluntary work in Norway, the contribution towards the care sector remains low, but stable (Folkestad et al., 2015). Current research in Norway indicates that 6% of the population carry out voluntary work within health, care and rescue work and 6% of the population do voluntary work within social services and abuse treatment (Fladmoe et al., 2018). These activities take place in different areas such as nursing homes and home care facilities (Jensen, 2015). However, recent research has found that volunteer activities are significantly more prevalent in nursing homes than in home care services (Skinner, 2018). Furthermore, *the nature* of these activities is characterised by social activities such as going to cafés, excursions, exercising and visiting services in addition to practical support (Agenda Kaupang AS, 2014; Jensen, 2015; Skinner et al., 2018; Solbjør et al., 2012). Likewise, healthcare professionals express different perspectives regarding voluntary contributions in institutional care (Engel, 2003). Engel (2003) observed that on the one hand, the professionals acknowledge the need for voluntary contributions regarding social activities, whilst on the other hand, they are afraid of being deprived of pleasant and less arduous care tasks. Even though voluntary organisations perform vital tasks for the public care services, 60% of the municipalities want to establish their own voluntary service (Abrahamsen, 2010).

As previously indicated, collaboration is already taking place between the public care services and voluntary organisations in Norway. However, current government policies and documents call for increased collaboration between the public care sector and voluntary organisations as well as an increase in the volume of voluntary work in the care sector (Ministry of Health and Care Services, 2013). A Norwegian national strategy describes goals to be achieved in the interaction between the voluntary sector and the public sector for the period 2015–2020 in the care services (Ministry of Health and Care Services, 2015). The strategy outlines a number of key measures to be implemented in collaboration between the care services and the third sector, of which three are of interest for the focus of this paper (Ministry of Health and Care Services, 2015). One measure emphasises interaction and collaboration between the voluntary sector and the municipality, including developing an overall, local voluntary policy. In this connection, the Ministry of Health and Care Services (2015) also wants to facilitate stronger collaboration with voluntary organisations in order to encourage more citizens to participate. The second measure of interest for this paper focuses on how to prepare actions and written agreements for the actors in order to create predictability and clarify responsibilities when volunteers contribute to the care sector. Finally, a third measure focuses on mobilising resources generally, for example, recruiting new volunteers but is also particularly directed towards the elderly, such as coordinating walking groups, dancing, visiting services and other social activities (Ministry of Health and Care Services, 2015).

To explore these challenges and expectations towards collaboration between voluntary organisations and care services in the municipalities, this paper utilises the theoretical concept of collaborative innovation and a theoretical framework on co-production. Together, they make up the theoretical toolbox used to interpret the empirical results on collaboration.

## Theoretical framework

### Collaborative innovation and co-production

Sørensen and Torfing (2011b) highlight the fact that if the right conditions exist, innovation in the public sector will be strengthened through multi-actor and interdisciplinary collaboration. Collaboration amplifies the exchange of information, knowledge, ideas and critical assessments and coordinates individual and collective actions, additionally co-creating solutions (Sørensen and Torfing, 2011a). Furthermore, the collaboration between different actors may increase resource efforts (Torfing et al., 2014). In order to manage this together, trust must exist among the collaborating actors (Sørensen and Torfing, 2011b). Trust is also important when discussing new ideas and suggestions in an open and unprejudiced way, and when giving and receiving responses related to the latter (Sørensen and Torfing, 2011b).

When different actors collaborate, institutional logics can be seen as shaping ‘the rules of the game’ in a given context (Thornton and Ocasio, 2008, p. 112). An institutional logics approach points to how institutions by representing different underlying logics of action, ‘shape heterogeneity, stability and change in individuals and organizations’ (Thornton and Ocasio, 2008, p. 103). Moreover, institutional logics are defined as ‘The socially constructed, historical patterns of cultural symbols and material practices, including assumptions, values, and beliefs, by which individuals and organisations provide meaning to their daily activity, organize time and space, and reproduce their lives and experiences’ (Thornton et al., 2012, p. 2). Hence, a collaboration between municipalities and voluntary organisations might be influenced and disrupted by different logics.

Concerning collaborative innovation, Bommert (2010) has described the principal features of this as follows ‘ […] the innovation process is opened up, that actors from within the organization, other organizations, the private and third sector and citizens are integrated into the innovation cycle (idea generation, selection, implementation and diffusion) from the earliest stage onwards’ (p. 16). Additionally, innovation is always contextual, what is new is new in a given context (Sørensen and Torfing, 2011b). The theoretical reasoning here is in line with the political recommendations in the aforementioned white papers, arguing for an expanded capacity if new actors (such as volunteers) are brought into the handling of ‘public tasks’. In this context, co-production is initiated.

Co-production is described as a ‘relationship between a paid employee of an organization and (groups of) individual citizens that requires a direct and active contribution from these citizens to the work of the organization’ (Brandsen and Honingh, 2016, p. 431). The concept of co-production originates from Elinor Ostrom in the 1970s (Parks et al., 1981). However, interest in co-production in mobilising citizen involvement through third sector organisations did not occur until 2000 (Bovaird and Loeffler, 2012). Co-production has been studied in different contexts and for different phenomena (Pestoff, 2012b), thus one must be specific when studying the concept, particularly regarding the different types of citizen participation and on which level the co-production occurs (Pestoff, 2012a; W. H. Voorberg et al., 2015). Of particular interest for this study, is the distinction between actions at the individual level and the organisational level. For example, you can fill roles as an individual or in close collaboration with others, for example, informal groups, or the collaboration can take place within the voluntary organisations or between the organisation and the care service (Pestoff, 2012b).

This study aims to identify the extent to which LTC units in Norwegian municipalities and voluntary organisations collaborate in the coordination of volunteer activities at the local, municipal level. In this study, LTC units are represented by nursing homes and home care districts. To provide empirical examples of how this collaboration takes place within care services in a Scandinavian context, this paper utilises the results from a survey targeting employees in public care services. The empirical part of the study presents data on the collaboration that takes place between the LTC units and voluntary organisations in coordinating volunteer activities. It focuses on how the LTC units and the voluntary organisations coordinate, separately or together, the volunteer activities provided in the care services.

## Methods

The study makes use of data from a survey conducted in 2015 in a sample of 50 Norwegian municipalities. The sample represented municipalities characterised as both urban and rural, small, medium-sized and large in all five regions of Norway.^1^ A team of four researchers developed a questionnaire. The questionnaire consisted of primarily closed-end, multiple-choice questions. It was based on a review of available literature on volunteering in the long-term care sector as well as qualitative interviews with informants from five different municipalities, carried out in Norway by one member of the research team during the spring of 2015.

### Data collection

The electronic questionnaire was distributed by email to the municipalities. In order to identify all the nursing homes and home care districts (here named as LTC units) in the sample, the research team contacted the municipalities prior to the distribution. Contacts in the municipalities identified 316 LTC units in total. Most of the units identified consisted of nursing homes and home care districts. However, integrated services were also identified. The email addresses of all the survey respondents were obtained. The respondents, one for each LTC unit, were employees working in or for each LTC unit, and also possessed special knowledge regarding volunteer activities provided there.

### Survey items

The respondents were asked to answer questions about volunteer activities organised within the LTC units in the previous four weeks. The respondents were also asked to state who coordinated the activity provided. Thus, in this paper, ‘coordinate’ is understood as ‘being responsible for organising the activity’.

The paper covers three main questions accompanied by several alternative answers, and the survey items used are presented in Table 1. For question three, the latter two alternatives, ‘unsure’ or ‘different coordination responsibility’, are left out of the analysis, as they do not identify coordination responsibility. The percentage answering these two alternatives varied from 0 to 35% between the six categories under study.

**Table 1:** Survey items used in the analysis

QUESTION 1
Which LTC unit do you answer for? Nursing home services Home care services Integrated services Other, describe:

QUESTION 2
Have any of the following volunteer activities been organised at your LTC unit during the last four weeks: Social activities – trips, social gatherings, etc.* Cultural activities for residents in nursing homes – music, dance, theatre, etc.* Cultural activities for recipients of home care services – music, dance, theatre, etc.* Library/reading services Physical activities and exercise* Visiting schemes Food-delivery for recipients of home care services* Practical help for recipients of home care services (e.g. snow clearing, food shopping etc.) Day centres operated by or with volunteers* Helplines, counselling services Transport/taking out Activities certified by the foundation ‘Joy of Life for the Elderly’ [Livsglede for eldre] Self-help groups (for anxiety, grief, loneliness, etc.) Other activities

QUESTION 3
Who coordinates volunteer activities provided by your LTC unit? The municipality* A Voluntary Organisation* A Voluntary Organisation and the municipality together* Several services with different coordination responsibilities Unsure

* Further analysed variables, see next section ‘Statistical analyses’.

### Statistical analyses

For each LTC unit, it was registered whether each voluntary activity was coordinated by the municipality alone, by the voluntary organisation alone, the two together or other arrangements. In relation to the initially envisioned forms of collaboration between public care services and voluntary organisations, and the division of tasks, the analyses focused on activities provided in both nursing homes and home care services. Moreover, two statutory and four non-statutory activities, corresponding to municipal responsibilities related to care services, were selected. The two selected statutory services were day centres, an activity offered to both recipients of home care services and residents in nursing homes and food delivery for recipients of home care services. The four remaining activities represented non-statutory care services and are: (1) cultural activities (music, dance, theatre, etc.) for residents in nursing homes, (2) cultural activities for recipients of home care services, (3) physical activities/exercising and finally (4) social activities (trips, social gatherings, etc.). The latter two activities are offered to both recipients of home care services and residents in nursing homes. Despite being a statutory public care service, the category ‘social activities’ is included here as a non-statutory service. The reason is that the municipalities do not report decisions about social activities to national registries according to the ‘Individual-based health and care statistics’ (IPLOS register) in Norway (Statistics Norway, 2016), which contains pseudonymous information about all care recipients in Norwegian municipalities.^2^

Altogether 244 LTC units responded to the survey, giving a response rate of 77.2%. The distribution between the LTC units was as follows: nursing homes 128, home care services 94, integrated services (both) 11 and other (assisted living facilities/day-care centre) 11^3^. There were no notable differences between the response rates among the nursing homes and home care units. Moreover, considering the spread of participating municipalities in terms of geography and size, the sample is judged to be fairly representative of the sector as a whole.

## Findings

### Responsible for statutory services

As presented in Table 2, the LTC units are mostly responsible for coordinating volunteer activities corresponding to public statutory services. For the two statutory services ‘day centres’ and ‘food-delivery (home care services)’, the percentage share coordinated by the LTC units corresponds to 95 and 62%, respectively. However, voluntary organisations are responsible for organising 15% of the ‘food-delivery (home care services)’ segment.

**Table 2.** Volunteer activities provided and responsible body

	Non-statutory services				Statutory services	
	Social activities (*n* = 101)	Cultural activities (nursing homes) (*n* = 73)	Physical activities/exercising (*n* = 52)	Cultural activities (home care services) (*n* = 37)	Food delivery (home care services) (*n* = 26)	Day centres (*n* = 19)
Municipality	(35%)	(49%)	(60%)	(22%)	(62%)	(95%)
Voluntary organisation	(42%)	(26%)	(19%)	(51%)	(15%)	0
Voluntary organisation and the municipality together	(24%)	(25%)	(21%)	(27%)	(23%)	(5%)
	100	100	100	100	100	100

Measured differences in proportions and differences in group means are statistically significant within a 99% confidence interval. All the analyses used R (The R Foundation for Statistical Computing, Vienna, Austria).

### Responsible for non-statutory services

Although voluntary organisations are better represented as coordinators for volunteer activities corresponding to non-statutory services, the LTC units also take responsibility for coordination in this category. For ‘social activities’ and ‘cultural activities (home care services)’, the proportion coordinated by voluntary organisations is highest (with 51 and 42%, respectively). But for ‘physical activities’ and ‘cultural activities (nursing homes)’, the LTC units dominate with 60 and 49%, respectively.

### Mixed coordination of volunteer activities provided

The findings in Table 2 show that LTC units and voluntary actors do share the coordinating of tasks in about one-quarter of cases for each activity, except for ‘day centres’ where collaboration is more limited (5%).

### Volunteer activities provided

Table 2 shows social activities like trips and social gatherings for the care receivers, both in home care services and in nursing homes, are the most frequently provided volunteer activities. Cultural activities in nursing homes are also frequently provided. This is expected and in line with earlier descriptions (Agenda Kaupang AS, 2014). However, volunteer activities solely for recipients of home care services are not so frequently provided.

## Discussion

When covering different types of collaboration involving citizen participation in public service provision, co-production can be used as a general term (Pestoff, 2012b).

There are currently two different co-production models presenting the various levels of co-production. Pestoff (2012b) focuses on the dyad between public agencies and voluntary actors on the organisational level. Osborne et al., (2016) on the other hand, pay attention to another dyad: the relationship between the helper and the user on the individual level, where services are performed. Agreements about co-production are most commonly formalised on the organisational level. This study utilises data from the survey to illustrate the co-production at the organisational level. Hence, this will be the focus of the discussion.

The background section launched two forms of collaboration between the public care services, that is, LTC units and voluntary organisations: the actors can have *separate tasks* or they can *share the same tasks*. This is related to both the organisation and performance of tasks.

The results from this study reveal three different ways of coordinating voluntary activities at the organisational level: How the responsibility for organising the voluntary activities is divided between the LTC unit and the voluntary organisation, or in collaboration between them. However, the findings in this study show an even more nuanced picture of the collaboration, as the coordination of activities does not fully correspond to the municipal statutory and non-statutory tasks as expected.

Concerning *separate tasks*, the findings confirm the expectations that LTC units take responsibility for coordinating statutory services. Consequently, voluntary organisations have limited responsibilities towards these services. Voorberg *et al.* (2017) illustrate that ‘state and governance traditions can explain why governments respond differently to similar challenges’ (p. 190). With its social-democratic regime (Esping-Andersen, 1990), Norway is characterised by a strong public sector, whereby the municipalities have a long tradition of being responsible for care service provision. By coordinating these activities, the municipalities perform their obligations since the municipalities in Norway are responsible for providing and managing care services (Ministry of Health and Care Services, 2011a).

Moreover, concerning *separate tasks*, voluntary organisations are expected to be better represented as coordinators, hence responsible for non-statutory services, than the municipalities. This study reveals that although voluntary organisations are responsible for some of the non-statutory services, the LTC units contribute largely in coordinating these services as well. This is a paradox since the municipalities are squeezed on resources and are recommended to increase the voluntary presence in the care services (Ministry of Health and Care Services, 2013). Due to the lack of an overall, local voluntary policy (Loga, 2012), the collaborative parties do not possess a common agreement on who does what activity. Despite the lack of such a policy, the municipalities are already in charge of providing and managing care services and they take the responsibility (Ministry of Health and Care Services, 2011a). The LTC units might also see this as a way to ensure quality and content in the services they provide to the care receiver (Ministry of Health and Care Services, 2003). This exemplifies why it is important to take the context of state and governance traditions into consideration, as Voorberg et al. (2017) argue.

Concerning *sharing the same tasks*, the study reveals a limited occurrence. These mixed responsibilities between LTC units and voluntary organisations happen in about one-quarter of the statutory and non-statutory activities described earlier. According to the theoretical framework, a successful collaboration can increase the total resources through co-production and stimulate innovations by bringing heterogeneity to the discussions and problem-solving (Torfing et al., 2014). However, this presupposes that collaboration actually occurs. Notably, this study reveals a limited occurrence.

Collaboration implies doing things together. The results of this study show that the actors, to some degree, coordinate volunteer activities together. Nevertheless, the LTC units control most of the coordination work, independently of statutory or non-statutory services, despite being in a situation with limited resources and demands for increased involvement from voluntary organisations. Thus, the LTC units’ dominance in the coordination can hardly be seen to optimise the conditions for co-production. This could indicate that the public sector, as a dominant actor, may have limited trust in the voluntary sector. Theories about collaboration and co-production pinpoint the need for trust (Sørensen and Torfing, 2011b); trust has to be present between actors when providing activities (Sørensen and Torfing, 2011a). Although this study does not contain data about trust or distrust among the actors, it is known that 60% of the municipalities want to establish their own voluntary services (Abrahamsen, 2010). Hence, there is a need for more knowledge about what creates trust and distrust in collaboration, and how to prevent competition between the actors from obstructing collaboration.

The municipalities and the voluntary organisations operate within different institutional logics. The different assumptions, values and beliefs each organisation brings into the collaboration must be taken into account (Thornton et al., 2012). These challenges seem to be underestimated by policymakers. This study reveals limited collaboration between the two actors at the organisational level. Hence, more knowledge is needed about how the different logics trigger competition instead of collaboration.

The concept of co-destruction has more or less been absent when scrutinising co-production in a public service context (Osborne et al., 2016). Plé and Chumpitaz Cáceresan (2010) define value co-destruction as an ‘interactional process between service systems that results in a decline in at least one of the systems’ well-being (which, given the nature of a service system, can be individual or organizational)’ (p. 431). In the co-production process, both the upside and the downside of value must be acknowledged (Echeverri and Skålén, 2011). The organiser may contribute in a negative way, and the interplay between the different actors can be destructive as well. As an example of co-destruction, voluntary contributions do not demand any professional training before co-producing services, which could lead to poor quality of services for care receivers. Furthermore, since the LTC units coordinate most of the activities, one can envisage threats to the volunteer spirit and autonomy, which could lead to an experience of co-destruction of services and co-destruction of value for the volunteers on an individual and organisational level.

## Conclusions

When voluntary organisations and LTC units collaborate in organising and providing volunteer activities in the Norwegian public care sector, a task division is revealed and there is a limited collaboration between the actors at the organisational level. Concerning the collaboration, there may be a lack of trust between the actors. The LTC units provide more than they are obliged to in terms of their statutory tasks. Although one main goal of the volunteer policy is to mobilise resources, and co-production has been advocated as a functional and innovative method of activating resources, there appears to be insufficient coordination to optimise the conditions for co-production. This study is conducted within a Nordic welfare regime (Esping-Andersen, 1990) with a strong public sector. This may leave little space for voluntary participation. However, other explanations, such as the different institutional logics, also have to be taken into account. The actors represent different assumptions, values and beliefs in a collaboration.

The findings of this study additionally point to a potential for reconsidering how future volunteer activities in collaboration between LTC units and voluntary organisations should be organised and carried out. Both policy documents and theoretical contributions operate at a high level of generalisation. There is, therefore, a need for studies that use a variety of approaches in order to provide knowledge about the interactions and the outcomes for the care receivers.

Only employees in the municipalities answered the questions in this study. It is worth noting that volunteers might have responded differently to the questions and hence should be included in future research. Other studies that focus on the individual level are also lacking. Moreover, another field that needs further research is the outcome of the co-production.

## Footnotes

1 The 5 regions in Norway are divided according to geographical location and represented 19 counties at the time the survey was conducted.

2 Since the statutory/non-statutory classification of social activities may be questioned, a sensitivity analysis was conducted, whereby means of proportions for non-statutory services excluding social activities were computed. Differences of proportions in the first comparison and differences of means of proportions in the second comparison were computed, together with 95% confidence intervals for these differences. The confidence intervals were computed by the bootstrap BC_a_ procedure (Efron & Tibshirani, 1993) using 10 000 bootstrap replications. This is a general procedure for computing confidence intervals that does not make particular distributional assumptions. Differences were regarded as significant if 0 was not included.

3 Measurements related to the coordination reported by LTC units that are active within the same municipality were not conducted, since the intention of the study was never to focus on differences between LTC units within the municipalities and/or differences between municipalities.
